# Food Hardness Modulates Behavior, Cognition, and Brain Activation: A Systematic Review of Animal and Human Studies

**DOI:** 10.3390/nu15051168

**Published:** 2023-02-25

**Authors:** Khaled Al-Manei, Leming Jia, Kholod Khalil Al-Manei, Elisande Lindström Ndanshau, Anastasios Grigoriadis, Abhishek Kumar

**Affiliations:** 1 Division of Oral Diagnostics and Rehabilitation, Department of Dental Medicine, Karolinska Institutet, 141 04 Huddinge, Sweden; 2 Division of Endodontics, Department of Restorative Dental Science, College of Dentistry, King Saud University, Riyadh 11545, Saudi Arabia; 3 Private Practice, Folktandvården Björkhagen, 120 08 Stockholm, Sweden; 4 Academic Center for Geriatric Dentistry, 112 19 Stockholm, Sweden

**Keywords:** chewing function, hard food, soft diet, rodents, cortical blood flow, functional magnetic resonance imaging

## Abstract

Food hardness is one of the dietary features that may impact brain functions. We performed a systematic review to evaluate the effect of food hardness (hard food versus soft food diet) on behavior, cognition, and brain activation in animals and humans (PROSPERO ID: CRD42021254204). The search was conducted on 29 June 2022 using Medline (Ovid), Embase, and Web of Science databases. Data were extracted, tabulated by food hardness as an intervention, and summarized by qualitative synthesis. The SYRCLE and JBI tools were used to assess the risk of bias (RoB) of individual studies. Of the 5427 studies identified, 18 animal studies and 6 human studies met the inclusion criteria and were included. The RoB assessment indicated that 61% of animal studies had unclear risks, 11% had moderate risks, and 28% had low risks. All human studies were deemed to have a low risk of bias. The majority (48%) of the animal studies showed that a hard food diet improved behavioral task performance compared to soft food diets (8%). However, 44% of studies also showed no differential effects of food hardness on behavioral tests. It was also evident that certain regions of the brain were activated in response to changes in food hardness in humans, with a positive association between chewing hard food, cognition performance, and brain function. However, variations in the methodologies of the included studies hindered the meta-analysis execution. In conclusion, our findings highlight the beneficial effects of dietary food hardness on behavior, cognition, and brain function in both animals and humans, however, this effect may depend on several factors that require further understanding of the causality.

## 1. Introduction

Chewing is one of the phylogenetically oldest functions of the stomatognathic system. The chewing function is accomplished by rhythmic movements of the jaw, which breaks up large pieces of food into smaller pieces, forming a soft, lubricated bolus that is safe for swallowing [1]. The central pattern generators in the brain stem initiate and generate jaw movement rhythms during chewing [2,3,4]. The jaw movements are also continuously modulated by the sensory inputs from peripheral receptors that signal the changing properties of the food during mastication. In particular, sensory information regarding the magnitude, direction, amplitude, and rate of tooth loading during tooth–food contact plays an integral role in the sensorimotor regulation of biting [5,6,7,8] and chewing behaviors [9]. Adaptation to the changing physical characteristics of the food is caused by changes in the muscle commands that alter jaw kinematics and chewing forces [7,9,10]. Further, the forces required to regulate the act of mastication and the various masticatory movements are not only controlled by the brainstem but also influenced by the motor cortex. Thus, the chewing function is a complex, semiautomatic, subconscious activity that can be controlled consciously depending on the demands of the task.

The impact of chewing on maxillofacial development, stomatognathic system balance, and central motor control are extensively documented in the literature [11,12]. Several experimental and epidemiological studies have investigated the relationship between chewing function and cognitive status or cerebral activation [13,14,15,16]. In particular human experimental studies have shown that short-term use of chewing gum results in increased regional cerebral blood flow and enhancement of cognitive function, such as working memory [15,16]. Evidence from the transcriptomic investigation has also suggested that chewing function may modify the functions of microglia in the brain, which in turn may affect the neuroimmune activity and cognitive function [17]. Overall, these studies suggest that chewing function is associated with cognitive health and may impact cognitive performance. 

A substantial rise in the number of people with cognitive impairment and dementia has led to several research initiatives aimed at identifying the associated modifiable risk factors [18]. Food and nutrition are considered one of the most significant modifiable risk factors impacting cognition [19]. Specific nutrients such as fatty acids, antioxidant vitamins, and vitamins, as well as foods such as fish, fruits, and vegetables, have been suggested to be protective against cognitive decline and dementia [20,21,22,23,24,25]. However, dietary features such as hardness, which are distinct from food and nutritional content may also have an impact on brain functions in general and cognition in particular [26]. 

It is shown that the ability to chew moderately hard food is associated with high-level functional capacity including the social role and intellectual abilities [27]. It is also suggested that brain activity may change depending on the strength of the movements in the oral and maxillofacial areas [28]. According to a positron emission tomography study, chewing with moderate bite forces causes changes in the internal carotid arterial blood flow with a subsequent increase in cortical blood flow [29]. Further, findings from a registry-based study suggested that the “inability to eat hard food” was associated with greater odds of cognitive impairment when controlled for confounding factors [14]. Research on rodents in particular has suggested that hard diets improve neurogenesis [30], while soft diets may influence behavior and increase vulnerability to mental disorders [31]. However, a number of studies that have examined functional brain activity during chewing function and other behavioral testing have been inconclusive in establishing a reasonable “cause–effect” relationship [32]. Therefore, the current systematic review aims to evaluate the literature on the effect of food hardness on behavior, cognition, and brain activation in animal models and humans. We hypothesized that animals fed with foods of relatively higher hardness would perform better in cognitive and behavioral tasks, along with greater activation in the areas of the brain responsible for these functions. Furthermore, we also hypothesized that eating/chewing food of moderate hardness would be associated with better performance in cognitive tests or higher activation of cognition-related brain regions in humans.

## 2. Materials and Methods

### 2.1. Protocol and Registration

The protocol of the current systematic review was registered at the International Prospective Register of Systematic Reviews (PROSPERO) with the identification number CRD42021254204. The current systematic review was reported according to the Preferred Reporting Items for Systematic Reviews and Meta-analyses (PRISMA-2020) statement guidelines [33]. The research question was created based on the PICOS scheme, namely: Population (healthy dentate human and animal subjects); Intervention (soft food); Comparison (hard food); Outcome (behavior, cognitive function, and brain activation); Studies (interventional and observational studies). A detailed description of PICOS and inclusion and exclusion criteria are presented in Table 1. 

### 2.2. Search Strategy

Two librarians conducted the literature search using three electronic databases: Medline (Ovid), Embase, and Web of Science Core Collection, from the beginning until the 29th of June 2022. Search terms including the relevant medical subject heading (MeSH) terms and keywords are summarized in Supplementary File S1. A Google Scholar search for the first 100 hits was also performed. Additionally, a manual search through the bibliographies of the included studies was applied to identify any additional relevant studies.

### 2.3. Eligibility Criteria

All the search results from the databases were tabulated by the EndNote 21 software. Duplicates (i.e., studies emerging in more than one database) were removed automatically by the built-in EndNote removal feature. The titles and abstracts of the studies were imported to Rayyan software (https://rayyan.qcri.org/welcome) and carefully screened to eliminate those that did not meet the scope of this systematic review. The full texts of the studies were retrieved and read for the inclusion criteria with no restrictions on the publication date or intervention duration. If information was missing, the corresponding authors were first contacted for clarification before excluding the studies. All the above processes were carried out by four independent reviewers (K.A.-M., J.L., E.L.N., and A.K.). Any disagreement between the reviewers over the screened or included studies was resolved with a mutual discussion with the other authors (K.K.A.-M. and A.G.). The inter-rater reliability values between reviewers ranged from 0.70 to 0.90 for the different variables collected. Additionally, only original research articles that have been peer-reviewed and published in peer-reviewed journals were included. 

### 2.4. Risk Bias Assessment of Included Studies

According to the study type/model, two critical appraisal tools were used to assess the quality and risk of bias of the included studies. For the animal studies, the SYRCLE’s risk of bias (RoB) tool was applied as suggested by the SYstematic Review Centre for Laboratory Animal Experimentation. The SYRCLE’s RoB tool is based on the Cochrane Collaboration’s RoB tool, which assesses the methodological quality and adapts for factors of bias that play a crucial role in animal studies. This tool consists of 10 items, covering 6 types of biases: selection bias, performance bias, detection bias, attrition bias, reporting bias, and other biases. The items were reported in the form of an inquiry to which the answer was “Yes; indicating low risk of bias”, “No; indicating high risk of bias”, or “Unclear; indicating item not reported”. Human studies were assessed through Joanna Briggs Institute critical appraisal tools (JBIs) for the trustworthiness, relevance, and results of published studies. JBIs consist of eight questions, scored “Yes”, “Unclear”, or “No”, with the same definition as SYRCLE’s scores. Scoring decisions were classified as (A) low risk of bias if ‘yes’ score ≥ 70%; (B) moderate risk of bias if ‘yes’ score = 50–69%; (C) high risk of bias (if yes) scored ≤ 49%. The assessment was performed by three independent reviewers (K.A.-M., J.L., and K.K.A.-M.), and any dispute was resolved through joint discussion with the other authors (E.L.N., A.G., and A.K.).

### 2.5. Data Collection and Management

The key characteristics of each included study were collected by four independent authors (K.A.-M., J.L., E.L.N., and K.K.A.-M.) and summarized in a spreadsheet (Microsoft Excel^®^, Albuquerque, NM, USA) with the following variables: author(s), publication year, country, title and journal name, study objective(s), study design, number of subjects (humans or animals) per a study, study groups and food hardness, consumption time, outcome measures and assessment methods, results including behavioral and radiographical findings, and conclusions. For the behavioral data, the end time point of the sequential behavioral tests was collected. In addition, radiographical data presented in text, tables, or graphical illustrations were also gathered. However, due to inconsistencies in the methodologies of the included studies (see limitations), quantitative data synthesis for meta-analysis could not be carried out as originally intended. 

## 3. Results

### 3.1. Study Selection

**Figure 1:**
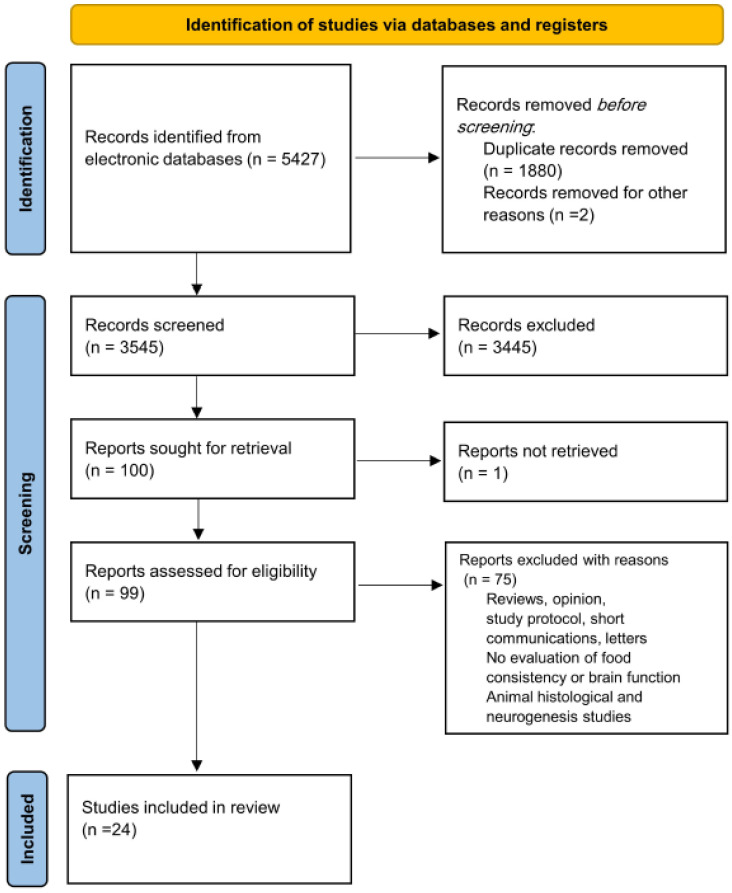
PRISMA flow diagram showing the screening and study selection.

The database search preliminarily resulted in 5427 studies and finally 3545 studies after removing the duplicates. Screening the titles/abstracts of the search yielded 100 eligible studies but 1 study could not be retrieved [34]. Full-text screening of the shortlisted studies resulted in a final list of 24 included studies. The PRISMA flowchart used to guide the selection of the studies is presented in Figure 1. 

### 3.2. Characteristics of the Included Studies

Eighteen studies were conducted in Japan, three in Brazil, one in Italy, and one study each in the USA and Canada. Of the 24 studies included in this systematic review, 18 were animal studies. The approaches used in the animal experiments focused on behavioral testing, including aspects of cortical orofacial motor representation of jaw and tongue muscles [35], spatial learning and cognitive (memory) function [31,36,37,38,39,40,41,42,43,44], olfactory function [30], daily activities [31,45], adaption to new environments [31], and anxiety-related [46,47] and food-seeking behaviors [47]. In addition, six cross-sectional human studies were included. The methods used in the human studies involved neuroimaging assessments [48,49,50] and a series of cognition scales [26,32,51]. Table 2 summarizes the characteristics of the studies included.

### 3.3. Animal Studies

#### 3.3.1. Characteristics of Laboratory Animal Species, Strains, Sex, and Age

Rodents such as mice and rats were used in the animal studies. All the studies reported the species and strains of the animals used. The specific animal species included Wistar rats [36,52], Sprague Dawley rats [35], Aβ-infused Wistar rats [38], C57BL mice [30,31,41,43,44,45,47,53], BALB/c mice [46], B6C3Fe-a/a mice [37], Albino Swiss mice [40,42,54], and SAMR1 and SAMP8 mice [39]. Among the different kinds of animal species, Wistar rats, Sprague Dawley rats, C57BL mice, BALB/c mice, B6C3Fe-a/a mice, and Albino Swiss mice are widely used inbred strains for routine animal tests [55]. SAMP8 and SAMR1 are senescent accelerated prone and senescence-resistant mice, respectively, that are usually used to study aging and age-related diseases [56]. Wistar Aβ-infused rats are an animal model for Alzheimer’s disease [57]. Further, five studies used only female rats or female mice as test animals. While ten studies used male rats or male mice as the test animals, two studies used both female and male rats or mice. In one study, the sex of the animals was not reported [41]. The age of the experimental mice or the rats was about 3 to 28 weeks when the experimental intervention (feeding either soft or hard food) was made. The detailed animal species, strains, sex, and age of included articles are presented in Table 2.

#### 3.3.2. Intervention: Food Hardness

Food hardness as an intervention was investigated in all the included studies. Seventeen studies used standard pelleted food as the hard diet and powder food as the soft diet. One study used the 1.5-fold autoclave-treated pelleted food as the (extra) hard diet, and pelleted food as the soft diet [41]. Soft food and hard food in the same study contained the same manufactured material with the same nutritional value. Nine studies reported detailed food supplier companies and specific diet types. The food suppliers included Oriental Yeast Co., Tokyo, Japan (n = 4); Clea, Tokyo, Japan (n = 1); Oriental Yeast Co., Osaka, Japan (n = 1); Sapporo, Japan (n = 1); Nihon Noson, Kanagawa, Japan (n = 2). However, nine studies did not report the supplier company of the animal food. All eighteen studies investigated the effect of constant hard food and soft food intervention on various animal behavioral tests. Further, four studies additionally investigated the effect of masticatory rehabilitation, which is a combination of dietary hardness interventions, such as hard food/soft food followed by soft food/hard food or hard food followed by soft food and hard food [42]. 

#### 3.3.3. Housing Conditions and Other Experiment Conditions

The housing conditions of the animals in each study were evaluated, including light, time, temperature, humidity, rearing device, and living space. Typically, test animals are given a twelve-hour light/dark cycle. The exact lighting hour reported in the included studies was from 05:00 ± 4 h in the morning to 17:00 ± 4 h in the evening. The temperature of the housing was within the range of 22 ± 3 °C, and the humidity was 57.5 ± 7.5%. Rearing devices, for example, standard plastic cages were reported in sixteen studies. The standard living space of rats and mice varied between studies depending on the number of animals. For instance, a 22 × 16 × 12 cm space was provided for one individual mouse [47], 30 × 20 × 14 cm for five to six mice [39,46], 32 × 39 × 16.5 cm for six mice [40], 32 × 45 × 16.5 cm for nine [42] or twelve mice [54], and 47 × 31 × 20 cm for two–three rats [52]. Two studies reported two specific environmental conditions for animal experiments (an impoverished cage and an enriched cage), where the enrichment cages were equipped with large spaces (100 × 50 × 50 cm for five mice), bridges, tunnels, running wheels, and toys [42,54]. 

#### 3.3.4. Behavioral Test Findings

**Figure 2:**
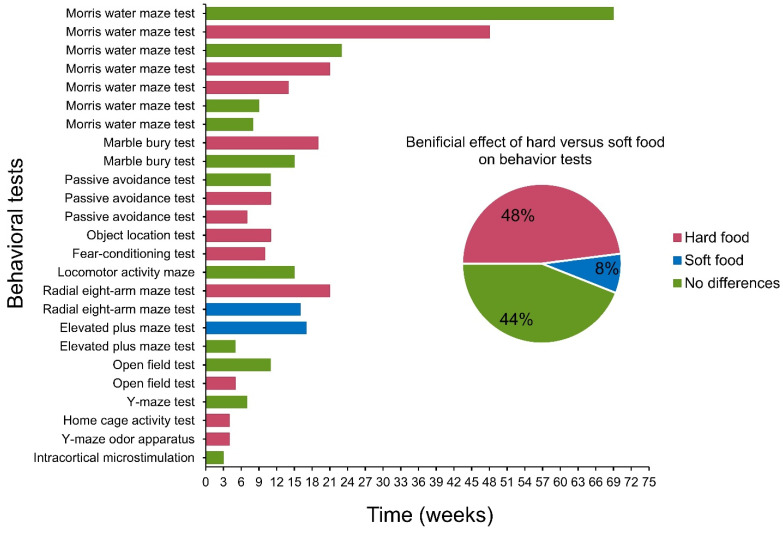
Effect of food dietary hardness on behavioral tests in animal studies.

The findings of the animal behavior testing of the fourteen included studies are summarized in Figure 2. Some studies had more than one behavioral test and the same tests were conducted at different time points. Fourteen different methods were applied for measuring behavioral outcomes. The behavioral measurements/outcomes included intracortical microsimulation (ICMS)-evoked electromyographic (EMG) activities [35], eight-arm radial maze [36,39], Morris water maze [31,37,40,41,42], passive avoidance test [38,43,44], Y-maze test [31], fear conditioning test [31], objective location test [44], Y-maze odor preference apparatus [30], home cage activity test [31], locomotor activity test [45], open field test [31], elevated plus maze [46], marble burying test, and food deprivation test (activity, temperature, and sleep) [47]. Four studies were not included in Figure 2 due to several factors such as sex differences [52], environmental enrichment [42,54], and the integrity of dentition [53]. The behavioral test of the other four studies included an open-field test [31,44,52,54] and a passive avoidance test in one study [53]. Most studies (48%) found that the hard food diet was beneficial for better performance in behavioral tests compared to the soft food diet (8%). However, 44% of animal studies also showed no differential effects of food hardness on the performance of the behavioral task. 

### 3.4. Human Studies

#### 3.4.1. Characteristics of Included Participants

Only six human studies with a cross-sectional design investigated the effects of food hardness on cognitive function and brain activation. Three studies looked at the correlation between chewing gum hardness and brain activation in young healthy individuals aged 20–32.5 years [48,49,50]. Three studies investigated the cognitive function of the elderly group aged 67–74 years [26,32,51]. Five studies recorded the sex of participants. The detailed characteristics of included participants are shown in Table 2.

#### 3.4.2. Intervention Approaches

Chewing gums of different hardness and two distinct questionnaires, a self-assessment of masticatory ability questionnaire and a brief-type questionnaire of self-administered dietary history, were used as intervention methods for the included studies. Three studies used chewing gums of different hardness, where the participants were asked to chew on gums with different hardness at different times [48,49,50]. In two included studies, the self-assessed masticatory ability was used as an index to assess the sequence and dynamics of mastication, and the participants were divided into three groups: (a) chews a variety of foods; (b) chews only slightly hard foods; (c) chews only soft or pureed foods [32,51]. One study used a brief-type, self-administered diet history questionnaire as an intervention and divided participants into four groups according to the hardness of the diet: (a) cooked rice; (b) green leafy vegetables; (c) dried fish; and (d) pork and beef [26]. 

#### 3.4.3. Brain Activation and Cognition Assessment Methods

Brain activation was objectively evaluated by using functional magnetic resonance imaging (fMRI) in three of the included studies [48,49,50]. Higher brain function and cognitive status were assessed by neuropsychological tests and cognition tests in three included studies [26,32,51]. Raven’s Colored Progressive Matrices (RCPM) test, the Verbal Paired Associates 1 (VerPA) task, the Visual Paired Associates 1 (VisPA) task (from the Wechsler Memory Scale-Revised Edition), and Block Design Subtest (from the Wechsler Adult Intelligence Scales-Third Edition) were the four neuropsychological tests. One study used the Tokyo Metropolitan Institute of Gerontology Index of Competence (TMIG-index) comprising three sublevels: instrumental self-maintenance, intellectual activity, and social role as an assessment method. Montreal Cognitive Assessment—Japanese version (MoCA-J) was used in the last included human study [26].

#### 3.4.4. Functional Magnetic Resonance Imaging (fMRI) and Cognition Test Findings 

One included study reported the effect of chewing gums with different hardness on regional brain activation, which was evaluated by fMRI [48]. Generally, chewing increased blood oxygenation level-dependent (BOLD) signals in the sensorimotor cortex, supplementary motor area (SMA), insula, thalamus, and cerebellum bilaterally. However, chewing hard gum resulted in lower cortical activation compared with moderately hard gum. Two fMRI studies in young adults demonstrated that selected regions of the brain are activated in response to changes in gum hardness [49,50]. The sensory input and motor output involved in changes in food hardness during chewing are probably linked in areas such as the SMA, dorsolateral prefrontal cortex (DLPFC), superior temporal gyrus (STG) of the left hemisphere, and the premotor area (PM) and inferior parietal lobule (IPL) of the right hemisphere.

A higher decline in brain function was significantly associated with impaired self-assessed masticatory function. Two included studies evaluated the global intellectual function and social role of community-dwelling persons after adjusting for several background factors and dental status [32,51]. Major factors contributing to dietary hardness like cooked rice, green leafy vegetables, dried fish and pork, and beef were positively associated with MOCA-J scores [26].

#### 3.4.5. Risk of Bias Assessment 

**Figure 3:**
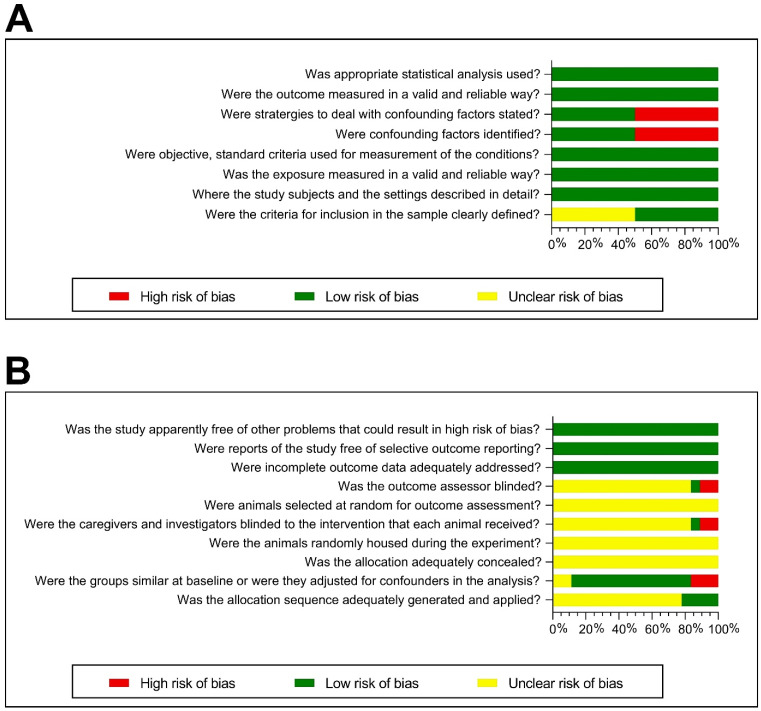
Summary of the risk of bias assessments of the included studies. (**A**) Risk of bias assessment for human cross-sectional studies (Joanna Briggs Institute critical appraisal tools). (**B**) Risk of bias assessment for animal studies (SYRCLE’s risk of bias tools).

The results of the risk of bias assessment for animal and human studies are shown in Figure 3A,B. Overall, eleven animal studies (Figure 3A) were classified as unclear risk, two studies as moderate risk, and five as low risk of bias. In terms of allocation sequence, four studies were found to have a low risk of bias, while the rest had an unclear risk of bias. Most studies had an unclear risk of bias concerning allocation concealment, caregiver blinding, and assessor blinding. Regarding baseline group similarity, nearly all studies had comparable animal populations by sex and age and were assessed as having a low risk of bias. Data concerning the allocation concealment and randomization of animal housing remained unclear. The risk of bias associated with the random selection of animals to assess outcomes was also judged as unclear for all the studies. The risk of dealing with incomplete data, selective outcome reporting, and other potential risk issues was rated as low in all studies. Three of the human studies included in the analysis had a moderate risk of bias, while the other three had a low risk. All human studies adequately described the study subjects and setting and were judged as having a low risk of bias (Figure 3B). However, in three of them, the inclusion criteria were unclear. Three studies rated the risk of identifying and managing confounders as high, while three rated it as low. With regards to the validity and reliability of exposure and outcome measures, statistical analyzes, and standardization of measures, all studies were found to be at low risk of bias.

## 4. Discussion

Dementia or other cognitive impairments are a major cause of disability and dependency among older people and pose a global challenge for health and social care [58]. Effective treatments or drugs for dementia and cognitive impairment are not yet widely available [59]. It is suggested that early intervention with the “right” foods, nutrients, and texture/hardness can delay the onset of cognitive impairment [60,61]. This systematic review examined the literature from animal and human studies on how food hardness impacts behavior, cognitive function, and brain activation. According to the results of this study, dietary hardness has a positive impact on behavior, cognition, and brain function in animals as well as humans, although this may depend on several factors. In particular, most studies showed that animals fed a hard food diet performed better in behavioral tests than animals fed a soft food diet. Further, results from human studies indicated that a higher decline in brain function was significantly associated with impaired subjective masticatory function, and food hardness was associated with higher activation of regions of the brain responsible for cognition. The main findings from this study are discussed in detail below. 

### 4.1. Effect of Diet Hardness on Cognitive Functions in Animals

Animal studies have indicated that masticatory impairment caused by tooth extraction or alteration of the occlusion by reducing the natural height of teeth causes chronic stress, and decreases learning ability, spatial memory, and hippocampal neurons [62,63]. However, the precise relationship between dietary hardness and cognitive function has not been evaluated. The results of this systematic review showed that food/diet hardness has a beneficial effect on behavior and cognitive function, although it may be affected by several factors. One such factor was the duration for which the animals were kept on either (hard or soft) diets. Accordingly, in all the eighteen animal studies included in the current systematic review, the animals (mice or rats) were fed with the hard or soft diet for several weeks. However, the timing of dietary intervention varied across the studies. Animals fed a consistent diet of hard food performed better in behavioral tests than those fed with soft foods [30,31,37,38,39,40,41,42,43,44,45,46,47,53,54]. Specifically, mice fed with a hard food diet had increased hippocampal volume compared with mice fed the soft food [41]. In contrast, animals fed with soft or powdered diets showed adverse symptoms [47], reduced working capacity [39,43], decreased spatial memory [44], and impaired learning ability [38]. This suggests that a hard food diet has a favorable/beneficial impact on cognitive function in animals, although it would be dependent on the duration of the intervention. 

### 4.2. Effect of Masticatory Rehabilitation on Cognitive Functions in Animals

Four of the included studies also examined the impact of masticatory rehabilitation (see, Intervention: Food Hardness, above), in which further investigations were carried out in a third independent group or the same group while examining the impact of the constant hard food/soft food intervention [30,31,42,54]. The experimental group was designed in two forms under masticatory rehabilitation conditions. Mice were fed a soft diet for several weeks and then switched to a hard diet until behavioral testing was performed, or mice were fed a hard diet, a soft diet, and then a hard diet for several consecutive weeks. One study showed that performance on a Y-maze odor preference apparatus decreased in the masticatory rehabilitation group compared to the hard diet group [31]. Another study found no difference between the hard food diet group and the masticatory rehabilitation group at different time points during several behavioral tests [54]. Two other studies showed no difference between the dietary rehabilitation, hard food diet, or soft food diet groups [30,31,42,54]. These findings suggest that the effect of masticatory rehabilitation on brain activation and cognitive function in animals remains uncertain. 

### 4.3. Effect of Diet Hardness on Cognitive Functions and Brain Activation in Humans

The findings from behavioral experiments in humans have shown interesting correlations between eating hard food (or the subjective ability to eat hard food) and neurocognitive performance [26,27]. In particular, these studies have shown a significant association between the consumption of hard food (or chewing ability) and performance in MoCA cognitive tests [26,51] and intellectual activity [51]. The correlation can also be bilateral, so people with poor cognitive function can also have poor mastication, resulting in poor food choices and avoiding foods that are difficult to consume. It is difficult to establish cause–effect relationships from correlation studies, so the underlying mechanisms must be explored [64].

The exact mechanisms in humans are unknown, but animal studies have provided some explanations. One of the potential mechanisms is that mastication influences memory processes by reducing endocrinologic and autonomic stress responses, leading to increased activity of the hippocampus and prefrontal cortex [65]. Further, it is also suggested that a soft diet reduces the synaptic density of the cortex and decreases the pyramidal hippocampal cell count which can influence cognitive functions [39]. Moreover, chewing hard food stimulates the secretion of gastrointestinal hormones and simulates the afferent fibers of the vagal nerve to release cholecystokinin in the brain which is important for the chemical processes of memory and learning [66,67]. These suggestions are further strengthened by the findings from magnetic resonance imaging data in humans that have confirmed the association between chewing hard food and the activation of memory centers [48,68]. Specifically, the imaging studies suggest that chewing hard food produced stronger BOLD signals and activation of different regions of the brain including the dorsal prefrontal cortex. However, studies have also reported weaker BOLD signals in the premotor cortical areas of the ascending parietal gyrus [50] when chewing hard food compared to soft food. While the premotor cortex and parietal gyrus are mainly responsible for controlling voluntary muscle movements and processing somatosensory information, respectively, the prefrontal cortex is responsible for cognitive functions. Therefore, it is suggested that masticatory function, particularly chewing hard foods, may have an active role in increasing cognitive processing. However, more studies with adequate sample sizes and thorough hypotheses are needed to explore the effect of food hardness on cognitive functions. 

### 4.4. Other Factors Affecting the Effect of Hard Food Diets on Brain Function

Food hardness can affect cognitive function and brain activation differently under certain conditions. Sex was the first investigated factor in the included studies. One study showed that feeding a soft diet enhances the performance in radial eight-arm maze learning in female rats but not in male rats [36]. Another study showed sex-related differences in visuospatial ability and neurogenesis indicators in rats fed with a pelleted diet [52]. Sex differences in cognitive decline have also been investigated in other human studies. Well-known sex differences in Alzheimer’s disease (AD) dementia include disproportionately higher prevalence and lifetime risk for developing AD dementia in women compared to men [69,70]. Moreover, there are some differences between men and women regarding the prevalence of behavioral and psychological symptoms [71]. Therefore, more caution is needed in the selection of animal species or study populations, as behavioral testing may have differential effects between sexes. 

Similarly, another factor is environmental enrichment which has been specially investigated in two of the included animal studies. The beneficial effects of enriched environments have long been established [72]. Typical animal-enriched environments involve access to larger, more stimulating environments, with the goal being for socialization and voluntary physical activity [73]. Several lines of evidence showed that enrichment housing conditions for rodents can ameliorate abnormal behaviors and enhance cognitive functioning [74,75]. As a result, the mice fed with hard food diets and dwelling in an enriched housing environment performed better in the behavioral tests [42,54]. Environmental enrichment research has also been extended to human studies. It has been applied as a treatment for neurodevelopmental disorders (NDDs) [76]. In the current systematic review, two studies reported that the participants were community-dwelling older individuals, while other studies did not report the housing conditions of the participants. However, it has been emphasized that the housing conditions of older people, especially those with dementia, can be an important factor in care [77,78]. However, it is still unclear how the interaction between the hard diet and enriched housing affects cognition, particularly in humans.

The interaction between dentition integrity and food hardness has been investigated in one study. It showed that mice with intact teeth given hard food or soft food perform better in the passive avoidance test than mice with their molars extracted [53]. This is in agreement with a previous report where people with tooth loss are more likely to have impaired cognitive test performance [79]. In addition, edentulous participants also faced a 1.54-fold higher risk of cognitive impairment and a 1.40-fold higher risk of being diagnosed with dementia [80]. Hence, further research is needed to discover whether hard food intervention can also have a positive effect on the participants with tooth loss.

### 4.5. Limitations and Implications

In the present study, a meta-analysis was not conducted since the selected studies are largely heterogeneous. Animal studies and human studies are different in terms of food intervention approaches and cognitive assessment methods. In animal studies, which vary in species, strains, ages, and periods, it is nearly impossible to quantify and standardize all parameters. Another form of heterogeneity in animal studies is the duration of the intervention. Different studies had different time points and periods of intervention. The time point at which the rodents start to feed on the hard/soft food diet (intervention), the duration of food intervention, and the age when the rodents took the behavioral tests are largely responsible for the vast heterogenicity. First, the duration of food intervention is typically divided into short-term, medium-term, and long-term durations. However, the definition of the intervention period varies from study to study. For example, one study defined nine weeks of intervention as short-term and 21 weeks of intervention as a long-term intervention [40]. Another study defined a short-term intervention as 23 weeks and a long-term intervention as 48 weeks [37]. The exact duration of hard food intervention is different, but the authors agreed that a longer duration of intervention tended to produce more beneficial effects than a short-term intervention. In addition, different studies’ results were influenced by multiple factors like those discussed above. Therefore, the results are synthesized by extracting the words and texts to combine the results (narrative synthesis) from multiple studies. The quality assessment of the included studies was evaluated to determine the bias and risks in the design, conduction, and outcome which provides a current overview of the quality of evidence. However, the radiographical and cognition questionnaire-based human studies and behavioral test-based animal studies provide relatively uncomprehensive evidence on the impact of hard food on brain activation and cognitive function. Therefore, clinical studies with more rigorous study designs and robust methods should be used, especially in human studies, in the future. 

## 5. Conclusions

The results of the current systematic review show that dietary hardness has a beneficial impact on behavior, cognition, and brain function in animals as well as humans, although it may be dependent on several factors. Moreover, the preliminary impression of the human studies suggests a higher decline in brain function was significantly associated with impaired masticatory function. In addition, food hardness was associated with increased activation of brain regions involved in cognition. The results of the study are inconclusive in establishing the cause–effect relationship, mainly due to the lack of human clinical trials and large heterogenicity in animal studies.

## Figures and Tables

**Table 1:** PICOs criteria for inclusion and exclusion of studies.

	Inclusion Criteria	Exclusion Criteria
Population (P)	Healthy dentate human and animal subjects	Children below 18 years
Intervention (I)	Powder food Soft chewing gums, soft pureed food, liquid/semisolid diet, cooked rice, green leafy vegetables	Food with no indication of softness
Comparison (C)	Pellet food Hard chewing gums, all kinds of food, only slightly hard food, dried fish, pork, and fish	Food with no indication of hardness
Outcomes (O)	Behavior, cognitive function, and brain activation (animal behavioral tests and human neuroimaging evaluation and cognition evaluation assessments)	No direct and clear findings of behavior, cognitive function, and brain activation (animal histological and neurogenesis studies)
Studies design (S)	Peer-reviewed, original studies published in the English language	All reviews (narrative or systematic), meta-analyses, study protocols, conference abstracts, letters to editors, commentaries, preprints, case reports, not peer-reviewed studies, and articles published in a language other than English

**Table 2:** Overview of the included studies.

A. Animal Studies								
No.	Authors (Year), Country	Study Design	Subjects	Age and Sex	Intervention and Comparison Groups	Consumption Time	Outcome Measures and Applied Methods	Main Findings
1	Endo et al. (1994) [36], Japan.	Animal: Experimental study.	Wistar-Imamichi rats (N = 62).	Age: 3 wk. Sex: 29 M and 33 F.	Intervention: SF (N = NA). Comparison: HF (N = NA).	16 wk.	Behavioral task: radial eight-arm maze test.	The number of correct choices in the last five–seven trials was greater in rats fed SF than in rats fed HF. Additionally, the number of correct choices was significantly greater in F compared to M.
2	Yamamoto and Hirayama (2001) [39], Japan.	Animal: Experimental study.	S-A mice (N = 29).	Age: 3 wk. Sex: 20 M.	Intervention: SF: (1) S-AMR1 (N = 5) and (2) S-AMP8 (N = 5). Comparison: HF: (1) S-AMR1 (N = 5) and (2): S-AMP8 (N = 5).	21 wk.	Behavioral task: radial eight-arm maze test.	Significant impairment in working memory performance resulting from SF feeding was recognized in both S-AMR1 and S-AMP8 mice.
3	Takase et al. (2005) [52], Japan.	Animal: Experimental study.	Wistar rats (N = 56).	Age:3 wk. Sex: 28 M and 28 F.	Intervention: SF (N = 28). Comparison: HF (N = 28).	7–11 wk.	Behavioral task: radial eight-arm maze test, open-field test.	No significant differences were observed in the behavioral task (the spatial ability) between rats fed SF or HF. In rats fed HF, M performed better than F in the radial 8-arm maze task.
4	Mitome et al. (2005) [45], Japan	Animal: Experimental study.	C57BL/6 mice (N = 54).	Age: 4 wk. Sex: 54 F.	Intervention: (1) SF (N = 18); (2) SETF (N = 18). Comparison: (3) HF (N = 18).	15 wk.	Behavioral task: locomotor activity test.	No significant differences were observed in the behavioral task (locomotor activity test) between mice fed SF of HF.
5	Tsutsui et al. (2007) [37], Japan.	Animal: Experimental study.	B6C3Fe-a/a mice (N = 109).	Age: 3 wk. Sex: 109 M.	Intervention: SF: (1) for 180 days (N = 26) and (2) for 360 days (N = 29). Comparison: HF: (1) for 180 days (N = 24) and for 360 days (N = 30).	23 and 48 wk.	Behavioral task: Morris water maze test.	No significant difference in the escape latency was found between the 180-day-old HF group and 180-day-old SF group. However, a tendency to prolong the escape latency was observed in the 360-day-old SF group compared with the 360-day-old HF group.
6	Kushida et al. (2008) [38], Japan.	Animal: Experimental study.	Wistar Aβ-infused rats (N = 38).	Age: 3 wk. Sex: 38 M.	Intervention: SF (N = 28). Comparison: HF (N = 28).	7 wk.	Behavioral task: passive avoidance test.	STL time of rats fed SF was significantly shorter than rats fed HF indicating that SF feeding impairs learning ability.
7	Avivi-Arbwe et al. (2010) [35], Canada.	Animal: Experimental study.	Sprague Dawley rats (N = 12).	Age: NA. Sex: 12 M.	Intervention: SF (N = 6). Comparison: HF (N = 6).	2–23 wk.	Behavioral task: ICMS-induced EMG recordings.	No significant differences between the HF and SF groups in orofacial motor representations of the jaw and tongue within the face-M1 and adjacent face-S1.
8	Frota de Almeida et al. (2012) [40], Brazil.	Animal: Experimental study.	Albino Swiss mice (N = 66).	Age: 3 wk. Sex: 66 F.	Intervention: SF (N = 30). Comparison: HF (N = 36).	9, 21, and 69 wk.	Behavioral task: Morris water maze test.	Escape latencies of 6-month-old mice fed HF were significantly shorter than age-matched mice fed SF. However, no significant changes in escape latencies were observed between SF and HF groups at the age of 3 months or 18 months.
9	Mendes et al. (2013) [42], Brazil.	Animal: Experimental study.	Albino Swiss mice (N = 222).	Age: 3 wk. Sex: 222 F.	Intervention: HF/SF (N = 62). Comparison: (1) HF (N = 92) and (2) HF/SF/HF (N = 68). Under two conditions: IE or EE and two ages: 6- and 18- Mon- old.	24 and 74 wk.	Behavioral task: Morris water maze test.	For learning rate, and independent of age and condition, (HF/SF) was associated with lower learning rate and performance values compared with control (HF) or masticatory rehabilitated (HF/SF/HF) mice. Similar findings in swim speed and distance traveled, 6-month-HF/SF traveled longer distances than 6-month-HF and 6-month-HF/SF/HF, but shorter than 18-month-HF/SF and 18-month-HF/SF/HF.
10	Akazawa et al. (2013) [41], Japan.	Animal: Experimental study.	C57BL/6 mice (N = NA).	Age: 6 wk. Sex: NA.	Intervention: SF (N = NA). Comparison: HF (N = NA).	14 wk.	Behavioral task: Morris water maze test.	Mice fed HF required significantly less time to reach the platform than mice fed SF.
11	Nose-Ishibashi et al. (2014) [31], Japan.	Animal: Experimental study.	C57BL6/J mice (N = 21–30).	Age: 3 wk. Sex: 21–30 M.	Intervention: SF (N = 7–10). Comparison: (1) SF/HF (N = 7–10) and (2) HF (N = 7–10).	4–10 wk.	Behavioral task: home cage activity test (4 wk.), elevated plus-maze test and open-field test (5 wk.), Y-maze test (7 wk.), Morris water maze test (8 wk.), fear conditioning test (10 wk.).	Elevated plus maze test, Y-maze test, Morris water maze test, and classical fear conditioning test did not show any differences in the SF and SF/HF as compared to the HF. In the open field test, the total distance of locomotion in 15 min was significantly greater in SF than in HF. In the home cage activity test, the SF showed significantly lower activity levels per day than the HF. No differences in the behavioral tests were noted between HF and SF/HF.
12	Okihara et al. (2014) [43], Japan.	Animal: Experimental study.	C57BL/6J mice (N = 14).	Age: 3 wk. Sex: 14 M.	Intervention: SF (N = 7). Comparison: HF (N = 7).	11 wk.	Behavioral task: passive avoidance test.	In the HF group, the latency 24 h after one trial training significantly increased compared with that of training, but not in the SF group indicating an impairment in memory.
13	Utsugi et al.(2014) [30], Japan.	Animal: Experimental study.	C57BL/6 mice(N = 131).	Age: 24–28 wk. Sex:131 F	Intervention: SF (N = 32). Comparison: (1) HF (N = 31); SF/HF (N = 43).	4 and 12 wk.	Behavioral task: Y-maze odor preference apparatus.	In the HF group and SF/HF group, the preference ratio significantly increased compared with the SF group after 4 wk.
14	Anegawa et al. (2015) [47], USA.	Animal: Experimental study.	C57BL/6J mice (N = 20).	Age: 3 wk. Sex: 20 M.	Intervention: SF (N = 10). Comparison: HF (N = 10).	15 wk. and 19 wk.	Behavioral task: marble burying test (15 wk.), food-deprivation test (19 wk.).	No significant difference in the marble burying test between SF and HF. SF induced attenuated diurnal sleep/wake rhythm. SF showed less enhancement of wake/locomotor activity compared to HF.
15	Takeda et al. (2016) [53], Japan.	Animal: Experimental study.	C57BL/6K mice (N = 48).	Age: 28 wk. Sex: 28 M	Intervention: SF (N = 12). Comparison: HF (N = 12). With two conditions: IT and ET.	4 wk. and 16 wk.	Behavioral task: passive avoidance test.	No significant difference in latency times between the groups in the acquisition trial after 4 wk. The latency time of the ET/SF group was shorter than the IT/HF group after 16 wk.
16	Fukushima-Nakayama et al. (2017) [44], Japan.	Animal: Experimental study.	C57BL/6J mice (N = 63).	Age: 3 wk. Sex: 63 M.	Intervention: SF (N = 32). Comparison: HF (N = 31).	11 wk.	Behavioral task: passive avoidance, object location tests, and open-field test.	The frequency to sniff the moving object was lower in the mice fed with SF than in HF, suggesting impaired spatial memory.
17	Mendes et al. (2019) [54], Brazil.	Animal: Experimental study.	Albino Swiss mice (N = 180).	Age: 3 wk. Sex: 180 F.	Intervention: HF/SF (N = 60). Comparison: (1) HF (N = 60) and (2) HF/SF/HF (N = 60). Under two conditions: IE or EE and two ages: 6-, 12-, and 18-month-old.	24 wk., 48 wk. and 75 wk.	Behavioral task: open field test.	Outcomes were significantly influenced by interactions between environment, age, and diet. The locomotor and exploratory activities in open field tasks declined with age and SF.
18	Yaoita et al. (2019) [46], Japan.	Animal: Experimental study.	BALB/c mice (N = 28–36).	Age: 3 wk. Sex: 28–36 M.	Intervention: SF (N = 10). Comparison: HF (N = 10).	17 wk.	Behavioral task: elevated plus-maze test.	SF increased the % of the open-arm time and the total number of arm entries, indicating that the mice have low anxiety, hyperactivity, and impulsive behaviors. The % of open-arm time in HF was increased by treatment with an anxiolytic agent but not in SF.
**B. Human Studies**								
**No.**	**Authors (Year), Country**	**Study Design**	**Subjects**	**Age and Sex**	**Intervention and Comparison Groups**	**Consumption Time**	**Outcome Measures and Applied Methods**	**Main Findings**
1	Onozuka et al. (2002) [48], Japan.	Human: Cross-sectional study.	Young adults (N = 17).	Age: 20–31 y. Sex: 10 M and 7 F.	Intervention: moderately HF (N = 17). Comparison: HF (N = 17).	half min	Radiographic evaluation: fMRI.	Chewing of HF produced a stronger BOLD signal than the chewing of moderately HF in the cerebellum, whereas the converse was true for the primary cortical area and non-primary cortical areas, except for the thalamus, in which no difference was seen between the types of the food.
2	Takahashi et al. (2007) [49], Japan.	Human: Cross-sectional study.	Young adults (N = 15).	Age: 22–32 y. Sex: 6 M and 7 F.	Intervention: change in the food hardness (N = 15). Comparison: hardest food (N = 15).	half min	Radiographic evaluation: fMRI.	With the changes in the food hardness, selective activation was noted in the SMA, DLPFC, and STG of the left hemisphere, and the PM and inferior parietal lobule.
3	Bracco et al. (2010) [50], Italy.	Human: Cross-sectional study.	Young adults (N = 10).	Age: 23–32 y. Sex: 7 M and 3 F.	Intervention: SF (N = 10). Comparison: HF (N = 10).	3 min	Radiographic evaluation: fMRI.	Chewing of HF produced a weaker BOLD signal than the chewing of SF in the primary motor and premotor cortical areas the ascending parietal gyrus of the primary somatic sensory cortex and non-primary cortical areas.
4	Moriya et al. (2011) [32], Japan.	Human: Cross-sectional study.	Old adults (N = 208).	Age: 70–74 y. Sex: 79 M and 129 F.	Intervention: (1) only SF (N = 20), (2) only slightly HF (N = 56). Comparison: chew all kinds of food (N = 132).	NA	Self-assessed chewing ability: 4 neuropsychological tests: (a) RCPM, (b) VerPA, (c) VisPA, (d) Block Design.	Significant and positive correlations were found between the RCPM test, the VerPA task, the Block Design test, and the ability to chew all kinds of food compared to the other groups.
5	Moriya et al. (2012) [51], Japan.	Human: Cross-sectional study.	Old adults (N = 366).	Age: 67–74 y. Sex: 138 M and 228 F.	Intervention: (1) only SF (N = 27), (2) only slightly HF (N = 94). Comparison: chew all kinds of food (N = 245).	NA	Self-assessed chewing ability: TMIG-Index: (a) instrumental self-maintenance, (b) intellectual activity, and (c) social role.	No significant differences in the instrumental self-maintenance scale among the three groups, but significant differences were found in the total score, intellectual activity, and social role.
6	Okubo et al. (2019) [26], Japan.	Human: Cross-sectional study.	Old adults (N = 635).	Age: 69–71 y. Sex: 292 M and 343 F.	Intervention: 38 food items (N = NA). Comparison: hardest food within the list (N = NA).	NA	Self-assessed chewing ability: MoCA-J Assessment.	Food hardness was positively associated with the MoCA-J score.

Abbreviations: y: years, M: male, F: female, SF: soft food, HF: hard food, SETF: soft food with maxillary and mandibular molar tooth extracted, min: minutes, fMRI: functional magnetic resonance imaging, BOLD: blood oxygenation level-dependent, SMA: supplementary motor area, DLPFC: dorsolateral prefrontal cortex, STG: superior temporal, PM: premotor area, RCPM: Raven’s Colored Progressive Matrices, VerPA: Verbal Paired Associates, VisPA: Visual Paired Associates, TMIG-Index: Tokyo Metropolitan Institute of Gerontology Index, MoCA-J: Montreal Cognitive Assessment—Japanese version, wk.: weeks, S-A: Senescence-accelerated, STL: Step-through latency, ICMS: intracortical micro-stimulation, EMG: electromyographic, IT: intact molar tooth, ET: extracted molar tooth, IE: impoverished environments, EE: enriched environments, h: hour, NA: not available.

## Data Availability

Available from the corresponding author on reasonable request.
